# Correlating physico-chemical properties of analytes with Hansen solubility parameters of solvents using machine learning algorithm for predicting suitable extraction solvent

**DOI:** 10.1038/s41598-024-68981-9

**Published:** 2024-08-13

**Authors:** Eman A. Mostafa, Mohammad Abdul Azim, Asmaa A. ElZaher, Ehab F. ElKady, Marwa A. Fouad, Fatma H. Ghazy, Esraa A. Radi, Mahmoud Abo El Makarim Saleh, Ahmed M. El Kerdawy

**Affiliations:** 1 Pharmaceutical Chemistry Department, Faculty of Pharmacy, Cairo University, Kasr El-Aini St., P.O. Box 11562, Cairo, Egypt; 2 Department of Pharmaceutical Chemistry, Faculty of Pharmacy, Newgiza University (NGU), Newgiza, km 22 Cairo-Alexandria Desert Road, Cairo, Egypt; 3 Health Minister’s Technical Office, Ministry of Health, Cairo, Egypt; 4 Egyptian Drug Authority, Giza, Egypt; 5 School of Pharmacy, College of Health and Science, University of Lincoln, Lincoln, UK

**Keywords:** Liquid–liquid extraction, Artificial Neural Network model, Hansen solubility parameters, Extraction solvent prediction, Model validation, Analytical chemistry, Bioanalytical chemistry

## Abstract

Artificial neural networks (ANNs) are biologically inspired algorithms designed to simulate the way in which the human brain processes information. In sample preparation for bioanalysis, liquid–liquid extraction (LLE) represents an important step with the extraction solvent selection is the key laborious step. In the current work, a robust and reliable ANNs model for LLE solvent prediction was generated which could predict the suitable solvent for analyte extraction. The developed ANNs model takes a set of chosen descriptors for the cited analyte as an input and predicts the corresponding Hansen solubility parameters of the suitable extraction solvent as a model output. Then, from the solvent combination’s appendix, the analyst can identify the proposed extraction solvents' combination for the cited analyte easily and efficiently. For the experimental validation of the model prediction capabilities, twenty structurally diverse drugs belonging to different pharmacological classes were extracted from human plasma. The extraction process was performed using the predicted extraction solvent combination for each drug and quantitively estimated by HPLC/UV methods to assess their extraction recovery. The developed LLE solvent prediction model is in- line with the global trend towards green chemistry since it limits the consumption of organic solvents.

## Introduction

Identification and quantitation of analytes in biological fluids, such as whole blood, blood plasma, serum, urine, and saliva represent the most common definition of bioanalysis of pharmaceuticals. Bioanalysis has multidisciplinary applications, for example, in hospitals, it is essential to ensure that patients are properly medicated and compliant. In addition, bioanalysis plays an important role during the drug development and clinical trial stages for pharmacokinetics, bioequivalence, bioavailability and toxicokinetic investigation as well as for ADME studies performed for the newly developed drugs^1,2^. Many factors influence the development of a robust bioanalytical method including the matrices of interest, the range over which analytes need to be measured, the physicochemical properties of the analyte as well as the analyte(s) extraction process which is a very crucial step in most analytical procedures, especially bioanalytical ones^3–5^. To ensure the robustness of the developed analytical method, simplification of the complex biological sample should be carried out while keeping the analytes that are present in extremely low levels^6^. Thus, sample preparation is one of the key steps in bioanalytical procedures which is considered the most challenging one as it consumes time and effort for selecting the best extraction solvent system that efficiently extract the target analyte with high recovery and purity^7^.

A key technique in sample preparation is liquid–liquid extraction (LLE) which can provide extracts with low levels of the co-extracted matrix material. LLE involves the distribution of sample components between two immiscible liquid phases where analytes must be soluble in the extraction solvent and have high partition coefficients in it, for that, several solvent combinations are to be tested to achieve the best analyte extraction recovery^8,9^. LLE process offers several advantages such as simplicity of the technique, high throughput, elimination of environmental hazards, and high selectivity of separation^10^. Despite the obvious advantages of LLE, some challenges are associated with the traditional LLE such as multistage time-consuming procedures and the consumption of large amounts of organic solvents^11,12^.

Hansen Solubility Parameters (HSPs) were developed by Charles M. Hansen^13,14^ to predict if one substance will dissolve in a solvent forming a solution. Each solvent is given three HSPs which measure the interaction energies between its molecules and the solute, viz, dD (the dispersion interaction energy), dP (the dipolar intermolecular forces energy) and dH (the hydrogen bond energy). HSPs are powerful descriptors for evaluating interactions of molecules and their solubility in different liquids, thus, they have been used for several purposes such as understanding the solubility and dispersion properties of carbon nanotubes and buckyballs. Furthermore, they are used for the fast selection of safer and cheaper solvent combinations where an undesirable solvent can be rationally replaced by a combination of more desirable solvents whose combined HSPs equal those of the original solvent^15–19^.

Artificial neural networks are a commonly used machine learning algorithm for data modeling which adapt to complex relations between input and output data on the basis of their supervised learning^20^. In any modeling study, model validation is a crucial step as it evaluates the predictive ability of the generated model and ensures the model's significance and that the model results are not merely due to a statistical chance^21^. Model validation is carried out using internal validation (e.g., cross-validation) as well as external validation which uses unseen test set to validate that the obtained model is not merely the result of a descriptor-target property chance correlation^22^.

The primary aim of the current study is to develop a robust and reliable LLE solvent prediction model based on the analyte’s descriptors to predict the suitable solvent, or solvent combination, for the extraction of this specific analyte from aqueous-based matrices e.g., plasma. The developed model should save time and effort facilitating the extraction process and reducing the number of trials and the volume of consumed organic solvents making liquid–liquid extraction easier, straightforward and more eco-friendly in line with green chemistry aspects. For the experimental validation of the model prediction capabilities, twenty structurally diverse drugs from different pharmacological classes were extracted from human plasma using the model predicted solvent combination for each drug and quantitively estimated by HPLC/UV methods to study their extraction recovery.

## Experimental

### Materials and reagents

The used drugs were supplied by different pharmaceutical companies. HPLC-grade acetonitrile and methanol were purchased from Sigma-Aldrich (Germany). Ortho-phosphoric acid, acetic acid and potassium hydroxide were supplied by EL-Nasr Pharmaceutical Chemicals Co., Egypt. Potassium dihydrogen phosphate and ammonium acetate were supplied by Sigma-Aldrich (Germany). Bi-distilled water was produced in-house (Aquatron Water Still, A4000D, UK). Membrane filters of size 0.22 μm were purchased from ChromTech (UK). Human blank plasma was obtained from the Holding Company for Biological Products and Vaccines (VACSERA, Egypt) and stored at – 70 °C.

### Instrumentation

The HPLC instrument (Agilent1100 series) was composed of an Agilent isocratic pump G1310A, Agilent UV–visible detector G1314A, an Agilent manual injector G1328B with (20 mL) injector loop and Inertsil ODS-3 column (5 µm, 150 mm × 4.6 mm). An Agilent syringe, (50 mL, USA) and a Powersonic 405 ultrasonic processor (Human Lab INC- Hwaseong city, Korea) were employed. The pH was adjusted by the addition of ortho-phosphoric acid or potassium hydroxide by means of a pH meter equipped with a glass electrode (Jenway, 3505, Essex, UK).

### LLE modeling

#### Dataset construction

The extraction data of sixty-three structurally diverse drug molecules belonging to different pharmacological classes covering a wide range of physicochemical properties were self-collected from literature. The selected extraction solvents were ethyl acetate, diethyl ether, tert-butyl methyl ether, and dichloromethane, whereas drugs extracted with toxic solvents (e.g., chloroform) were excluded. The values of HSPs of the solvents were obtained from Dr Manuel Díaz de los Ríos, Director of Derivatives Division, ICIDCA^23^.

#### Drawing structures and molecular descriptors calculation

Molecular Operating Environment (MOE, 2020.0901) software was used for all the molecular modeling studies. Canonical SMILES of the sixty-three drugs were imported from PubChem^24^ into the MOE which were then converted into 3D structures. Energy minimization was performed for the built compounds until a RMS gradient of 0.05 kcal mol^−1^ Å^−2^ with MMFF94x force field and the partial charges were automatically calculated. MOE molecular mechanics descriptors were calculated for each compound and RapidMiner 7.1.000 Basic Edition^25^ was used to remove low variance descriptors using *Remove Useless Attributes* operator as they add no additional information to the model ''redundant descriptors'', this left a pool of 301 descriptors. Based on the relation of the different descriptors to the target parameters ''Hansen solubility parameters'', we found that dipole moment (Dipole), Van der Waals volume Å^3^ (Vdw_vol), Van der Waals energy (E_vdw), and log octanol/water partition coefficient (logP(o/w)) are the most important descriptors.

#### Training set and test set generation

The selected 63 drugs were split manually in a random manner into a training set of 48 molecules and an external test set of 15 molecules such that the test set maintains the same distribution of Hansen solubility parameters (HSPs) in the original dataset by keeping the ratio of the different solvents in the training and test sets equals to the original dataset (Supplementary Table S1^26–73^ and Supplementary Table S2^74–88^ in Supplementary File).

#### LLE model generation

MATLAB (version: 7.12.0.635) (R2011a) was used for generating the ANN models. Mean Absolute Error (MAE) is the model evaluation metric used to describe the average model performance. Linear Layer (design) was used in the ANN model generation.

#### Model validation

To assess the prediction ability and the robustness of the generated models, the developed model was validated using:

- Internal validation: this was carried out using leave-20%-out cross-validation (CV_L20%O_) in which the training set was split into five subsets and training and test subsets were chosen such that each point appears in the test subset once. Five ANN models were generated using linear layer design network.
- External validation: This was carried out by using the generated model to predict Hansen solubility parameters for the independent test set. This should be a direct simulation of the real case scenario which requires the prediction of new compounds (unseen by the model).

### Experimental validation

**Figure 1:**
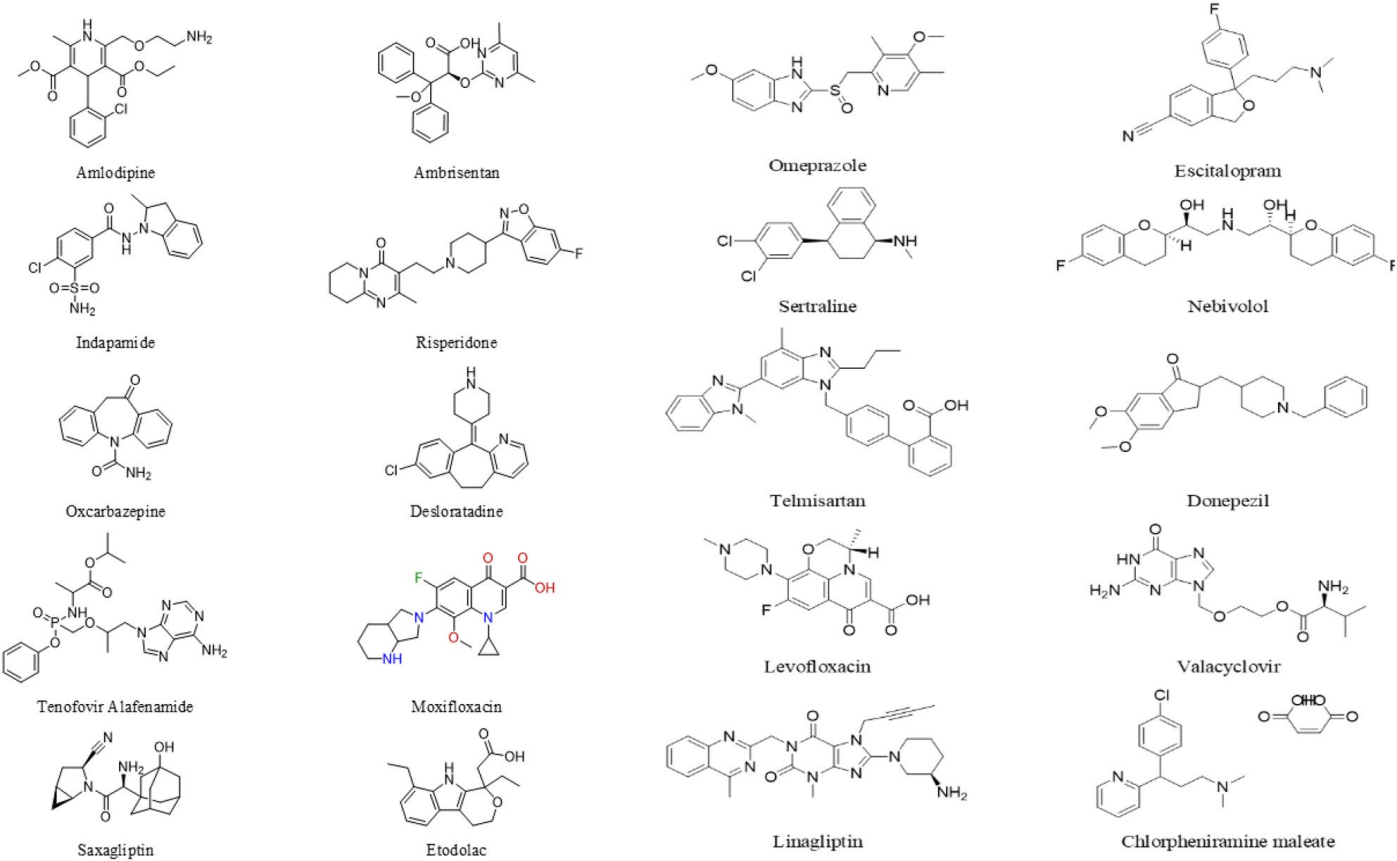
Chemical structures of the investigated drugs.

To test the generated model in a real case scenario, experimental validation of the model prediction was carried out. The developed ANN model was applied on twenty structurally diverse drugs from different pharmacological classes (Fig. 1) to predict their suitable extraction solvent combinations.

#### Prediction of the HSPs of the extraction solvent combination

First, the model’s four descriptors of the 20 drugs were calculated using MOE (Supplementary Table S3 in Supplementary File) and then ANN linear layer design model was applied on them to predict the HSPs of the solvent combinations to be used to extract each drug and using the solvent combination appendix (Supplementary Table S4 in Supplementary File) the corresponding solvent mixture for each drug was determined based on its predicted HSPs.

#### Determination of the solute recovery from spiked plasma using the predicted solvent combinations

The twenty drugs were extracted from spiked human plasma using the predicted solvent combinations. Various mobile phases and chromatographic conditions were used for the separation and quantitation of those drugs using HPLC/UV methods (Supplementary Table S5 and Supplementary Fig. S1 in Supplementary File). Selectivity of the developed chromatographic methods was confirmed by the absence of any interfering peaks from plasma samples at the retention times of the investigated drugs (Supplementary Fig. S2 in Supplementary File).

### Preparation of standard solutions

Stock solutions (1 mg/ml) were prepared by dissolving each drug in the appropriate HPLC-grade solvent (water, methanol or acetonitrile) and stored at 4 °C nominal. These stock solutions were diluted with a mixture of methanol and water (50: 50, *v/v*) to attain the required working solutions (100, 200 and 300 μg/ml).

### Preparation of human plasma samples and analyte extraction

Plasma samples (0.5 ml) containing the analyte were vortexed for 30 s. The extraction solvent mixture was added to the spiked plasma and blank samples. Samples were vortexed for 1.5 min, centrifuged at 4500 rpm for 10 min. The clear supernatant was transferred into a clean Wassermann tube then evaporated to dryness at 45 °C under the stream of Nitrogen then dried extract was reconstituted with 100 μl of the mobile phase.

### Procedure for extraction recovery calculations

The recovery following the sample preparation using the LLE model was evaluated by comparing the mean peak area of three extracted samples of low, medium, and high concentrations to the mean peak area of three plain standards of equivalent concentrations. Six replicates for each concentration were performed with the established extraction procedure.

### Ethical approval

This article does not contain any studies with human participants or animals performed by any of the authors.

## Results and discussion

**Figure 2:**
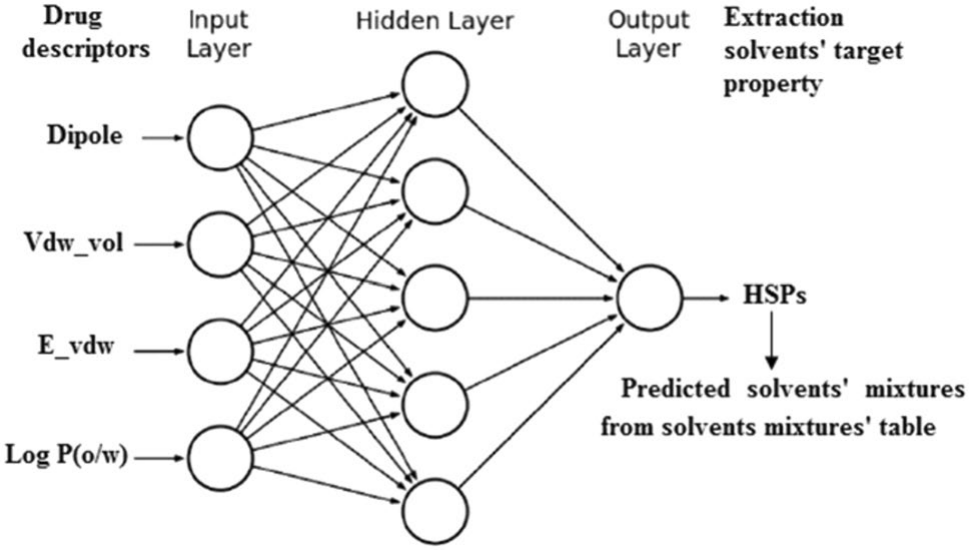
The developed ANN model structure.

A correlation between some of the molecular mechanical descriptors of the drugs, dipole moment, Van der Waals volume, and log octanol/water partition coefficient and the target property (HSPs) of the extraction solvents using ANN was performed. The selection of the descriptors was based on showing high mutual solubility intercorrelation. The target property (HSPs) are physicochemical parameters that are commonly used to estimate the form of interactive forces that cause material compatibility. The HSP assumes that cohesive energy (E) can be divided into three parts: atomic dispersion (Ed), molecular dipolar interactions (Ep), and hydrogen-bonding interactions (Eh). ANNs are a type of computer programs that can be taught to mimic relationships in data sets. After the ANN has been ‘trained,' it can be used to predict the outcome of a new set of input data, such as a different composite system. Linear Layer (design) was used in the ANN model generation (Fig. 2). The generation was done using a custom script written on MATLAB (version: 7.12.0.635) (R2011a).

The mean absolute error of a model represents the mean of the absolute values of the individual prediction errors on the overall instances in the dataset. Each prediction error is the difference between the predicted value and the true value for the instance.

$$MAE=\frac{\sum_{i=1}^{n}\left|{\widehat{y}}_{i -} {y}_{i}\right|}{n}$$

where ŷ_i_ is the predicted value, y_i_ is the true value, and n is the sample size.

### MAE of internal validation

MAE of Hansen solubility parameters was found to be: 0.77 ± 0.48 for Hansen D, 1.19 ± 0.87 for Hansen P, and 1.12 ± 0.46 for Hansen H. The MAE of CV was calculated by absolute subtracting the predicted values from reported ones then divided by the number of the training set (Supplementary Table S6 in Supplementary File).

### MAE of external validation

MAE of Hansen solubility parameters was found to be: 0.79 ± 0.56 for Hansen D, 1.14 ± 0.97 for Hansen P, and 1.23 ± 0.35 for Hansen H. the MAE of external validation was calculated by absolute subtracting the predicted values from reported ones then divided by the number of the test set (Supplementary Table S7 in Supplementary File).

### Predicted HSPs of the investigated drugs and the predicted solvents' combinations

The predicted Hansen solubility parameters of the twenty drugs were obtained from the application of the developed ANN model on those drugs. For better extraction recovery results, the use of extraction solvents’ combination is recommended than the use of a single extraction solvent. Supplementary Table S5 in Supplementary File shows the Hansen solubility parameters of different combinations of the four extraction solvents used in the developed model with different ratios. The fraction of ratio of each solvent has been multiplied to its Hansen solubility parameters then HSP values for both solvents have been summed giving the HSPs for the solvents' combination. By visual inspection of the solvents' combinations' table and the predicted HSP values obtained from the model, one or two solvent combinations could be selected that have HSP values close to the predicted values obtained from the prediction model (Table 1).

**Table 1:** Predicted Hansen solubility parameters and extraction solvents' mixtures of the investigated drugs using linear layer design model with their extraction recovery.

Drug	Hansen D	Hansen P	Hansen H	Predicted solvents' mixture (1)	Predicted solvents' mixture (2)	Extraction recovery*
Amlodipine	15.398	4.814	5.879	Ethyl acetate: TBME (60:40)*	Diethyl ether: Dichloromethane (60:40)	92.73 ± 0.34%
Ambrisentan	15.904	5.533	6.250	Dichloromethane: TBME (50:50)	Ethyl acetate: Dichloromethane (90:10)*	55.93 ± 0.45%
Risperidone	15.453	4.901	5.981	Ethyl acetate: TBME (60:40)	Dichloromethane: TBME (30:70)*	85.29 ± 0.35%
Indapamide	15.557	5.024	6.208	Ethyl acetate: TBME (70:30)	Ethyl acetate: Diethyl ether (80:20)*	93.29 ± 0.48%
Tenofovir alfenamide	15.045	4.336	5.807	Ethyl acetate: TBME (30:70)	Ethyl acetate: Diethyl ether (50:50)*	71.95 ± 0.40%
Oxcarbazepine	16.129	5.812	6.553	Ethyl acetate: Dichloromethane (80:20)*	Dichloromethane: TBME (60:40)	88.78 ± 0.67%
Desloratadine	15.776	5.330	6.118	Dichloromethane: TBME (40:60)*	Diethyl ether: Dichloromethane (50:50)	98.63 ± 0.41%
Saxagliptin	15.554	5.021	6.322	Ethyl acetate: Diethyl ether (70:30)*	Ethyl acetate: TBME (70:30)	69.45 ± 0.60%
Moxifloxacin	15.845	5.450	6.389	Ethyl acetate: Diethyl ether (90:10)*	Dichloromethane: TBME (50:50)	86.22 ± 0.69%
Etodolac	15.262	4.595	5.764	Ethyl acetate: TBME (40:60)*	Ethyl acetate: Diethyl ether (50:50)	83.02 ± 0.31%
Omeprazole	15.626	5.120	6.177	Ethyl acetate: Diethyl ether (90:10)	Ethyl acetate: TBME (80:20)*	98% ± 0.45%
Escitalopram	15.324	4.696	5.723	Ethyl acetate: TBME (50:50)	Diethyl ether: Dichloromethane (60:40)*	87% ± 0.41%
Sertraline	15.598	5.075	5.917	Diethylether:Dichloromethane (50:50)	Dichloromethane: TBME (30:70)*	91% ± 0.48%
Nebivolol	15.708	5.257	6.141	Ethyl acetate: Diethyl ether (90:10)*	Dichloromethane: TBME (40:60)	87% ± 0.30%
Telmisartan	14.902	4.158	5.035	Diethyl ether: TBME (10:90)	Ethyl acetate: Diethyl ether (30:70)*	53% ± 0.48%
Donepezil	15.423	4.857	5.824	Ethyl acetate: TBME (50:50)	Diethyl ether: Dichloromethane(60:40)*	66% ± 0.56%
Levofloxacin	16.215	5.962	6.752	Ethyl acetate: Diethyl ether (90:10)	Diethyl ether: Dichloromethane (50:50)*	61% ± 0.24%
Valacyclovir	15.569	5.033	6.414	Ethyl acetate: TBME (70:30)*	Ethyl acetate: Diethyl ether (80:20)	92% ± 0.49%
Linagliptin	15.611	5.140	6.098	Ethyl acetate: Diethyl ether (90:10)*	Ethyl acetate: TBME (80:20)	90% ± 0.36%
Chlorpheniramine maleate	15.122	4.433	5.633	Ethyl acetate: TBME (30:70)*	Ethyl acetate: Diethyl ether (40:60)	94% ± 0.32%

*Extraction recovery is the average recovery of three concentrations for each drug where each concentration was repeated six times.

*TBME tertiary-Butyl methyl ether.

### Recovery of the investigated drugs

Recovery of each drug was performed by comparing the results obtained from the analysis of plasma spiked with three different concentrations to non-extracted samples of equivalent concentrations (Table 1).

## Conclusion

A robust and validated LLE solvent prediction model which helps in predicting the organic extraction solvents' combinations for different drugs from aqueous-based matrices was built and validated. This was performed by making a correlation between some of the molecular mechanical descriptors of the drugs and the target property (HSPs) of the extraction solvents using ANN. Assessment of the prediction ability and the robustness of the generated model has been performed by internal and external validation. The generated ANN model has been applied on twenty drugs from different pharmacological classes. The extraction process of the investigated drugs was performed using the predicted extraction solvents' combination for each drug and quantitively estimated by HPLC/UV methods to study their extraction recovery. Good extraction recoveries were achieved. Therefore, bioanalysis could be much easier and more eco-friendly with the aid of the developed LLE solvent prediction model. The generated ANN model can be continuously improved by adding more input data to get more prediction capabilities.

### Supplementary Information

Supplementary Information.

## Data Availability

The authors declare that the data supporting the findings of this study are available within the paper and its Supplementary Information files. Should any raw data files be needed in another format they are available from the corresponding author upon reasonable request. Source data are provided with this paper.
