# Supplementary material for: Correlating physico-chemical properties of analytes with Hansen solubility parameters of solvents using machine learning algorithm for predicting suitable extraction solvent
Source: Sci Rep. 2024 Aug 13;14:18741. doi: 10.1038/s41598-024-68981-9 (PMC11322549; doi:10.1038/s41598-024-68981-9)
Supplement: Supplementary file 1 — Supplementary Information. [file 41598_2024_68981_MOESM1_ESM.docx]

**A Machine Learning Algorithm for predicting the suitable Liquid-Liquid Extraction Solvent and its Application in Drug Extraction from Human Plasma**

Eman A. Mostafa^a, *^, Mohammad Abdul Azim^a^, Asmaa A. ElZaher^a^, Ehab F. ElKady^a^, Marwa A. Fouad^a,b^, Fatma H. Ghazy^a^, Esraa A. Radi^c^, Ahmed M. El Kerdawy^a,d^

^a^ Pharmaceutical Chemistry Department, Faculty of Pharmacy, Cairo University, Kasr El-Aini St., Cairo, P.O. Box 11562, Egypt

^b^ Department of Pharmaceutical Chemistry, School of Pharmacy, Newgiza University (NGU), Newgiza, km 22 Cairo–Alexandria Desert Road, Cairo, Egypt

^c^ Health minister's technical office, Ministry of Health, Cairo, Egypt

^d^ School of Pharmacy, College of Science, University of Lincoln, Lincoln, United Kingdom

* Corresponding author:

*e-mail address*: [eman.saleh@pharma.cu.edu.eg](mailto:eman.saleh@pharma.cu.edu.eg)

h[ttps://orcid.org/0000-0003-0570-5731](https://mc.manuscriptcentral.com/aoac_jaoac)

***Table (S1): Training set of the developed ANN model***

| **Log P(o/w)** | **E_vdw** | **Vdw_vol** | **Dipole** | **Hansen H** | **Hansen P** | **Hansen D** | **Solvent** | **Drug** |
| --- | --- | --- | --- | --- | --- | --- | --- | --- |
| -0.73 | 12.51 | 168.37 | 0.6068 | 7.1 | 7.3 | 17.0 | Dichloromethane | 6-Mercaptopurine [26] |
| 2.02 | 46.44 | 719.12 | 1.0197 | 7.2 | 5.3 | 15.8 | Ethyl acetate | Boceprevir [27] |
| 4.82 | 80.62 | 663.63 | 1.4671 | 7.2 | 5.3 | 15.8 | Ethyl acetate | Buprenorphine [28] |
| -0.22 | 5.63 | 258.66 | 1.2708 | 4.6 | 2.9 | 14.5 | Diethyl ether | Busulfan [29] |
| 3.49 | 27.73 | 411.89 | 2.0391 | 5 | 4.3 | 14.8 | TBME | Celecoxib [30] |
| 3.12 | 93.80 | 1029.49 | 1.9542 | 5 | 4.3 | 14.8 | TBME | Clarithromycin [31] |
| 5.87 | 50.24 | 494.33 | 1.1496 | 7.2 | 5.3 | 15.8 | Ethyl acetate | Clemastine [32] |
| 1.04 | 34.45 | 535.76 | 0.9354 | 7.2 | 5.3 | 15.8 | Ethyl acetate | Clindamycin [33] |
| 3.38 | 48.09 | 412.32 | 0.1832 | 7.1 | 7.3 | 17.0 | Dichloromethane | Cyclizine [34] |
| 5.49 | 76.81 | 1006.36 | 2.3138 | 5.0 | 4.3 | 14.8 | TBME | Daclastasvir [35] |
| 1.78 | 36.38 | 419.58 | 0.1505 | 7.2 | 5.3 | 15.8 | Ethyl acetate | Dexlansoprazole [36] |
| 4.35 | 37.15 | 397.84 | 9.3262 | 7.2 | 5.3 | 15.8 | Ethyl acetate | Diclofenac sodium [37] |
| 5.06 | 40.06 | 401.56 | 0.8425 | 5.0 | 4.3 | 14.8 | TBME | Diethylstilbesterol [38] |
| 3.69 | 69.14 | 579.81 | 1.3283 | 7.1 | 7.3 | 17.0 | Dichloromethane | Dihydroetorphine [39] |
| 3.26 | 60.95 | 556.10 | 0.9222 | 5.0 | 4.3 | 14.8 | TBME | Diltiazem [40] |
| 3.78 | 59.70 | 556.84 | 0.9491 | 4.6 | 2.9 | 14.5 | Diethyl ether | Doxapram [41] |
| 2.91 | 46.01 | 409.90 | 0.5466 | 7.1 | 7.3 | 17.0 | Dichloromethane | Doxylamine [42] |
| 4.10 | 24.68 | 338.21 | 0.7992 | 7.2 | 5.3 | 15.8 | Ethyl acetate | Efavirenz [43] |
| -1.46 | 21.15 | 339.54 | 1.7351 | 5.0 | 4.3 | 14.8 | TBME | Entecavir [44] |
| 3.06 | 73.07 | 573.91 | 0.7758 | 7.1 | 7.3 | 17.0 | Dichloromethane | Etorphine [45] |
| 7.46 | 62.04 | 592.86 | 0.8358 | 5.0 | 4.3 | 14.8 | Ethyl acetate | Finasteride [46] |
| -1.12 | 31.12 | 358.87 | 1.1939 | 5.0 | 4.3 | 14.8 | TBME | Fluconazole [47] |
| 1.50 | 49.22 | 570.38 | 1.0789 | 4.6 | 2.9 | 14.5 | Diethyl ether | Glipizide [48] |
| 1.67 | 79.77 | 942.60 | 1.0507 | 5.0 | 4.3 | 14.8 | TBME | Indinavir sulfate [49] |
| 6.14 | 90.35 | 899.85 | 2.1981 | 5.0 | 4.3 | 14.8 | TBME | Itraconazole [50] |
| 4.03 | 63.83 | 666.49 | 2.0670 | 7.2 | 5.3 | 15.8 | Ethyl acetate | Ketoconazole [51] |
| 5.11 | 52.72 | 637.66 | 1.4015 | 5.0 | 4.3 | 14.8 | TBME | Lacidipine [52] |
| 3.40 | 54.61 | 521.93 | 0.8548 | 7.1 | 7.3 | 17.0 | Dichloromethane | Levocetrizine [53] |
| 1.42 | 63.52 | 636.73 | 0.6592 | 7.2 | 5.3 | 15.8 | Ethyl acetate | Sitagliptin [54] |
| 3.00 | 39.67 | 370.58 | 1.1887 | 7.1 | 7.3 | 17.0 | Dichloromethane | Lorazepam [55] |
| 1.64 | 20.26 | 381.16 | 1.2738 | 7.2 | 5.3 | 15.8 | Ethyl acetate | Lornoxicam [56] |
| 2.14 | 57.13 | 511.87 | 0.5915 | 5.0 | 4.3 | 14.8 | TBME | Methylprednisolone [57] |
| 1.71 | 35.32 | 400.96 | 0.9197 | 7.1 | 7.3 | 17.0 | Dichloromethane | Metoclopramide [58] |
| 2.27 | 52.34 | 432.31 | 1.2838 | 4.6 | 2.9 | 14.5 | Diethyl ether | Metolazone [59] |
| -1.52 | 27.62 | 401.33 | 0.9466 | 7.2 | 5.3 | 15.8 | Ethyl acetate | Mitomycin C [60] |
| 3.37 | 53.01 | 558.02 | 1.2061 | 4.6 | 2.9 | 14.5 | Diethyl ether | Mitragynine [61] |
| 3.29 | 38.23 | 318.96 | 0.6196 | 5.0 | 4.3 | 14.8 | TBME | Naproxen [62] |
| 2.47 | 56.73 | 551.44 | 0.9263 | 4.6 | 2.9 | 14.5 | Diethyl ether | Paliperidone [63] |
| 1.05 | 20.97 | 225.35 | 0.7508 | 7.2 | 5.3 | 15.8 | Ethyl acetate | Phenylpropanolamine [64] |
| 3.29 | 39.05 | 467.99 | 0.5167 | 4.6 | 2.9 | 14.5 | Diethyl ether | Pioglitazone [65] |
| 1.77 | 49.94 | 487.44 | 1.9555 | 7.2 | 5.3 | 15.8 | Ethyl acetate | Prednisolone [66] |
| 6.37 | 29.89 | 429.36 | 0.8112 | 5.0 | 4.3 | 14.8 | TBME | Tegaserod [67] |
| -2.72 | 16.51 | 212.24 | 0.5809 | 7.2 | 5.3 | 15.8 | Ethyl acetate | Temozolomide [68] |
| 7.67 | 70.83 | 756.68 | 0.5377 | 4.6 | 2.9 | 14.5 | Diethyl ether | Tipranavir [69] |
| 5.35 | 80.79 | 671.83 | 0.7660 | 4.6 | 2.9 | 14.5 | Diethyl ether | Verapamil [70] |
| 4.72 | 133.13 | 1099.76 | 2.0793 | 7.1 | 7.3 | 17.0 | Dichloromethane | Vincristine [71] |
| 5.01 | 115.04 | 1059.72 | 1.4715 | 5.0 | 4.3 | 14.8 | TBME | Vinorelbine [72] |
| 4.31 | 47.23 | 548.96 | 0.1975 | 5.0 | 4.3 | 14.8 | TBME | Zofenopril [73] |

***Table (S2): Test set of the developed ANN model***

| **Log P(o/w)** | **E_vdw** | **Vdw_vol** | **Dipole** | **Hansen H** | **Hansen P** | **Hansen D** | **Solvent** | **Drug** |
| --- | --- | --- | --- | --- | --- | --- | --- | --- |
| 4.55 | 124.69 | 1097.18 | 1.67 | 7.2 | 5.3 | 15.8 | Ethyl acetate | Rifampicin [74] |
| 2.82 | 75.47 | 662.11 | 1.32 | 7.2 | 5.3 | 15.8 | Ethyl acetate | Ivabradine [75] |
| 3.20 | 55.37 | 520.75 | 0.32 | 7.2 | 5.3 | 15.8 | Ethyl acetate | Hydroxyzine [76] |
| -0.85 | 8.383 | 129.88 | 0.77 | 7.2 | 5.3 | 15.8 | Ethyl acetate | 5-Fluorouracil [77] |
| 4.34 | 44.02 | 403.48 | 0.85 | 7.2 | 5.3 | 15.8 | Ethyl acetate | Fenofibric Acid [78] |
| 1.71 | 29.81 | 297.65 | 0.22 | 5.0 | 4.3 | 14.8 | TBME | Phenobarbital [79] |
| 1.08 | 59.87 | 498.48 | 2.04 | 5.0 | 4.3 | 14.8 | TBME | Dolutegravir [80] |
| 2.58 | 39.38 | 357.77 | 1.23 | 5.0 | 4.3 | 14.8 | TBME | Nimesulide [81] |
| 3.26 | 66.87 | 556.10 | 1.48 | 5.0 | 4.3 | 14.8 | TBME | Isradipine [82] |
| 3.32 | 99.65 | 1025.36 | 1.48 | 5.0 | 4.3 | 14.8 | TBME | Digoxin [83] |
| 3.08 | 50.74 | 544.13 | 1.28 | 4.6 | 2.9 | 14.5 | Diethyl ether | Nimodipine [84] |
| 2.15 | 51.74 | 515.01 | 1.69 | 4.6 | 2.9 | 14.5 | Diethyl ether | Betamethasone [85] |
| 3.84 | 43.13 | 334.64 | 0.30 | 4.6 | 2.9 | 14.5 | Diethyl ether | Tolfenamic acid [86] |
| 4.16 | 67.75 | 753.75 | 1.09 | 7.1 | 7.3 | 17.0 | Dichloromethane | Prochlorperazine maleate [87] |
| 3.38 | 49.59 | 412.32 | 0.35 | 7.1 | 7.3 | 17.0 | Dichloromethane | Ebastine [88] |

***Table (S3): Investigated drugs with their calculated descriptors***

| **logP(o/w)** | **E_vdw** | **vdw_vol** | **dipole** | **Drug** |
| --- | --- | --- | --- | --- |
| 2.44 | 47.75 | 526.43 | 0.4708 | Amlodipine |
| 3.46 | 64.84 | 518.26 | 1.1719 | Ambrisentan |
| 2.79 | 52.22 | 551.24 | 1.3265 | Risperidone |
| 1.83 | 38.52 | 431.50 | 1.6292 | Indapamide |
| 1.48 | 41.57 | 608.17 | 1.7746 | Tenofovir alfenamide |
| 1.96 | 47.34 | 338.16 | 0.8419 | Oxcarbazepine |
| 3.43 | 51.28 | 428.75 | 0.4199 | Desloratadine |
| 0.85 | 34.64 | 427.41 | 2.0635 | Saxagliptin |
| 1.89 | 56.85 | 510.59 | 1.7921 | Moxifloxacin |
| 3.73 | 32.30 | 400.54 | 0.5483 | Etodolac |
| 1.32 | 42.12 | 445.25 | 0.6931 | Omeprazole |
| 4.40 | 42.99 | 458.09 | 0.4280 | Escitalopram |
| 5.46 | 47.47 | 400.29 | 0.8207 | Sertraline |
| 3.22 | 58.11 | 521.28 | 1.2856 | Nebivolol |
| 8.49 | 72.56 | 730.27 | 0.4707 | Telmisartan |
| 4.146 | 55.40 | 549.61 | 0.9228 | Donepezil |
| 0.68 | 58.58 | 452.82 | 1.7552 | Levofloxacin |
| -0.87 | 28.07 | 403.90 | 1.6812 | Valacyclovir |
| 1.42 | 63.89 | 636.73 | 0.9223 | Linagliptin |
| 4.19 | 46.47 | 556.92 | 1.1582 | Chlorphineramine maleate |

***Table (S4): solvents' mixtures table***

| **Solvent Mixtures** | **Hansen D** | **Hansen P** | **Hansen H** |
| --- | --- | --- | --- |
| Ethyl acetate and Dichloromethane (10:90) | 16.9 | 7.1 | 7.1 |
| Ethyl acetate and Dichloromethane (20:80) | 16.8 | 6.9 | 7.1 |
| Ethyl acetate and Dichloromethane (30:70) | 16.6 | 6.7 | 7.1 |
| Ethyl acetate and Dichloromethane (40:60) | 16.5 | 6.5 | 7.1 |
| Ethyl acetate and Dichloromethane (50:50) | 16.4 | 6.3 | 7.2 |
| Ethyl acetate and Dichloromethane (60:40) | 16.3 | 6.1 | 7.2 |
| Ethyl acetate and Dichloromethane (70:30) | 16.2 | 5.9 | 7.2 |
| Ethyl acetate and Dichloromethane (80:20) | 16.0 | 5.7 | 7.2 |
| Ethyl acetate and Dichloromethane (90:10) | 15.9 | 5.5 | 7.2 |
| Ethyl acetate and Diethyl ether (10:90) | 14.6 | 3.1 | 4.9 |
| Ethyl acetate and Diethyl ether (20:80) | 14.8 | 3.4 | 5.1 |
| Ethyl acetate and Diethyl ether (30:70) | 14.9 | 3.6 | 5.4 |
| Ethyl acetate and Diethyl ether (40:60) | 15.0 | 3.9 | 5.6 |
| Ethyl acetate and Diethyl ether (50:50) | 15.2 | 4.1 | 5.9 |
| Ethyl acetate and Diethyl ether (60:40) | 15.3 | 4.3 | 6.2 |
| Ethyl acetate and Diethyl ether (70:30) | 15.4 | 4.6 | 6.4 |
| Ethyl acetate and Diethyl ether (80:20) | 15.5 | 4.8 | 6.7 |
| Ethyl acetate and Diethyl ether (90:10) | 15.7 | 5.1 | 6.9 |
| Ethyl acetate and Methyl-t-butyl ether (10:90) | 14.9 | 4.4 | 5.2 |
| Ethyl acetate and Methyl-t-butyl ether (20:80) | 15.0 | 4.5 | 5.4 |
| Ethyl acetate and Methyl-t-butyl ether (30:70) | 15.1 | 4.6 | 5.7 |
| Ethyl acetate and Methyl-t-butyl ether (40:60) | 15.2 | 4.7 | 5.9 |
| Ethyl acetate and Methyl-t-butyl ether (50:50) | 15.3 | 4.8 | 6.1 |
| Ethyl acetate and Methyl-t-butyl ether (60:40) | 15.4 | 4.9 | 6.3 |
| Ethyl acetate and Methyl-t-butyl ether (70:30) | 15.5 | 5.0 | 6.5 |
| Ethyl acetate and Methyl-t-butyl ether (80:20) | 15.6 | 5.1 | 6.8 |
| Ethyl acetate and Methyl-t-butyl ether (90:10) | 15.7 | 5.2 | 7.0 |
| Diethyl ether and Dichloromethane (10:90) | 16.8 | 6.9 | 6.9 |
| Diethyl ether and Dichloromethane (20:80) | 16.5 | 6.4 | 6.6 |
| Diethyl ether and Dichloromethane (30:70) | 16.3 | 6.0 | 6.4 |
| Diethyl ether and Dichloromethane (40:60) | 16.0 | 5.5 | 6.1 |
| Diethyl ether and Dichloromethane (50:50) | 15.8 | 5.1 | 5.9 |
| Diethyl ether and Dichloromethane (60:40) | 15.5 | 4.7 | 5.6 |
| Diethyl ether and Dichloromethane (70:30) | 15.3 | 4.2 | 5.4 |
| Diethyl ether and Dichloromethane (80:20) | 15.0 | 3.8 | 5.1 |
| Diethyl ether and Dichloromethane (90:10) | 14.8 | 3.3 | 4.9 |
| Dichloromethane and Methyl-t-butyl ether (10:90) | 15.0 | 4.6 | 5.2 |
| Dichloromethane and Methyl-t-butyl ether (20:80) | 15.2 | 4.9 | 5.4 |
| Dichloromethane and Methyl-t-butyl ether (30:70) | 15.5 | 5.2 | 5.6 |
| Dichloromethane and Methyl-t-butyl ether (40:60) | 15.7 | 5.5 | 5.8 |
| Dichloromethane and Methyl-t-butyl ether (50:50) | 15.9 | 5.8 | 6.1 |
| Dichloromethane and Methyl-t-butyl ether (60:40) | 16.1 | 6.1 | 6.3 |
| Dichloromethane and Methyl-t-butyl ether (70:30) | 16.3 | 6.4 | 6.5 |
| Dichloromethane and Methyl-t-butyl ether (80:20) | 16.6 | 6.7 | 6.7 |
| Dichloromethane and Methyl-t-butyl ether (90:10) | 16.8 | 7.0 | 6.9 |
| Diethyl ether and Methyl-t-butyl ether (10:90) | 14.8 | 4.2 | 5.0 |
| Diethyl ether and Methyl-t-butyl ether (20:80) | 14.7 | 4.0 | 4.9 |
| Diethyl ether and Methyl-t-butyl ether (30:70) | 14.7 | 3.9 | 4.9 |
| Diethyl ether and Methyl-t-butyl ether (40:60) | 14.7 | 3.7 | 4.8 |
| Diethyl ether and Methyl-t-butyl ether (50:50) | 14.7 | 3.6 | 4.8 |
| Diethyl ether and Methyl-t-butyl ether (60:40) | 14.6 | 3.5 | 4.8 |
| Diethyl ether and Methyl-t-butyl ether (70:30) | 14.6 | 3.3 | 4.7 |
| Diethyl ether and Methyl-t-butyl ether (80:20) | 14.6 | 3.2 | 4.7 |
| Diethyl ether and Methyl-t-butyl ether (90:10) | 14.5 | 3.0 | 4.6 |

***Table (S5): Chromatographic conditions of the investigated drugs***

| **Flow rate** | **Retention time** | **Mobile phase** | **Wave length** | **Drug** |
| --- | --- | --- | --- | --- |
| 1.4 ml/min | 3.735 min | 50 mM potassium dihydrogen phosphate buffer: acetonitrile (65: 35, v/v, adjusted with 0.01 N aqueous solution of NaOH to pH = 6) | 237 nm | Amlodipine |
| 1.4 ml/min | 5.398 min | 30 mM ammonium acetate buffer: acetonitrile (50: 50, v/v, adjusted with 0.01 N aqueous solution of acetic acid to pH = 4) | 215nm | Ambrisentan |
| 1 ml/min | 3.088 min | 10 mM ammonium acetate buffer: acetonitrile (40: 60, v/v, adjusted with 0.01 N aqueous solution of acetic acid to pH = 5.8) | 280 nm | Risperidone |
| 1.4 ml/min | 3.440 min | 20 mM potassium dihydrogen phosphate buffer: acetonitrile: methanol (40: 35: 25, v/v, adjusted with 0.01 N aqueous solution of NaOH to pH = 6.6) | 215nm | Indapamide |
| 1 ml/min | 2.872 min | 20 mM potassium dihydrogen phosphate buffer: methanol (30: 70, v/v, adjusted with 0.01 N aqueous solution of NaOH to pH = 5) | 260 nm | Tenofovir alfenamide |
| 1.5 ml/min | 6.70 min | H2O: methanol: acetonitrile (55: 40: 5, v/v) | 225 nm | Oxcarbazepine |
| 1 ml/min | 4.263 min | 20 mM potassium dihydrogen phosphate buffer: methanol (30: 70, v/v, adjusted with 0.01 N aqueous solution of NaOH to pH = 7) | 254 nm | Desloratadine |
| 1.4 ml/min | 4.906 min | 20 mM potassium dihydrogen phosphate buffer: acetonitrile (85: 15, v/v, adjusted with 0.01 N aqueous solution of o-phosphoric acid to pH = 4.5) | 218 nm | Saxagliptin |
| 0.8 ml/min | 3.239 min | 20 mM potassium dihydrogen phosphate buffer: methanol: triethylamine (40: 60: 1, v/v, adjusted with 0.01 N aqueous solution of o-phosphoric acid to pH = 2.8) | 290 nm | Moxifloxacin |
| 2 ml/min | 9.384 min | 20 mM potassium dihydrogen phosphate buffer: methanol: acetonitrile: triethylamine (40: 40: 20: 1.5, v/v, adjusted with 0.01 N aqueous solution of o-phosphoric acid to pH = 5.5) | 254 nm | Etodolac |
| 1 ml/min | 3.3 min | Methanol: Distilled water (75:25, v/v) | 304 nm | Omeprazole |
| 1 ml/min | 3.6 min | 50 mm Buffer: Acetonitrile (65:35, v/v, adjusted with 0.01 N aqueous solution of NaOH to pH = 6.3) | 270 nm | Telmisartan |
| 1 ml/min | 3.5 min | 50 mm Buffer: Acetonitrile (70:30, v/v, adjusted with 0.01 N aqueous solution of NaOH to pH = 6.8) | 239 nm | Linagliptin |
| 1 ml/min | 4.3 min | 20 mm Buffer: Acetonitrile: Methanol: Triethylamine (45:35:20:0.1, v/v, adjusted with 0.01 N aqueous solution of o-phosphoric acid to pH = 4). | 282 nm | Nebivolol |
| 1 ml/min | 4.6 min | 20 mm Buffer: Methanol: Triethylamine (45:45: 0.5, v/v, adjusted with 0.01 N aqueous solution of o-phosphoric acid to pH = 3.5). | 268 nm | Donepezil |
| 1 ml/min | 3.8 min | 20 mm Buffer: Acetonitrile: Methanol (50:25:25, v/v, adjusted with 0.01 N aqueous solution of NaOH to pH= 5.8). | 240 nm | Escitalopram |
| 1.4 ml/min | 3.7 min | 20 mm Buffer: Acetonitrile: Methanol: Triethylamine (45:35:20:0.01, v/v, adjusted with 0.01 N aqueous solution of o-phosphoric acid to pH = 4) | 220 nm | Sertraline |
| 1 ml/min | 3.3 min | 50 mm Buffer: Acetonitrile: Triethylamine (75:30:0.01, v/v, adjusted with 0.01 N aqueous solution of o-phosphoric acid to pH= 3). | 230 nm | Chlorphineramine Maleate |
| 0.5 ml/min | 4.6 min | 50 mm Buffer: Acetonitrile: Methanol: Triethylamine (70: 20: 10: 0.01, v/v, adjusted with 0.01 N aqueous solution of NaOH to pH= 6.5). | 253 nm | Valacyclovir |
| 0.8 ml/min | 3.3 min | Methanol: Distilled water: Triethylamine (70:30:0.01, v/v, adjusted with 0.01 N aqueous solution of NaOH to pH= 7.4). | 294 nm | Levofloxacin |

***Table (S6): predicted and reported HSPs of the 5 subsets used in CV***

| **Drug** | **Predicted HSPs** | | | **Reported HSPs** | | |
| --- | --- | --- | --- | --- | --- | --- |
| 6-Mercaptopurine | 15.779 | 5.3119 | 6.621 | 17.0 | 7.3 | 7.1 |
| Boceprevir | 14.389 | 3.4138 | 5.185 | 15.8 | 5.3 | 7.2 |
| Buprenorphine | 15.773 | 5.351 | 5.894 | 15.8 | 5.3 | 7.2 |
| Busulfan | 15.104 | 4.371 | 6.180 | 14.5 | 2.9 | 4.6 |
| Celecoxib | 15.007 | 4.245 | 5.774 | 14.8 | 4.3 | 5.0 |
| Clarithromycin | 14.803 | 4.037 | 5.280 | 14.8 | 4.3 | 5.0 |
| Clemastine | 15.241 | 4.574 | 5.515 | 15.8 | 5.3 | 7.2 |
| Clindamycin | 14.834 | 4.020 | 5.664 | 15.8 | 5.3 | 7.2 |
| Cyclizine | 15.673 | 5.178 | 5.943 | 17.0 | 7.3 | 7.1 |
| Daclastasvir | 14.066 | 2.989 | 4.650 | 14.8 | 4.3 | 5.0 |
| Dexlansoprazole | 15.404 | 4.708 | 5.870 | 15.8 | 5.3 | 7.2 |
| Diclofenac sodium | 16.213 | 6.559 | 8.042 | 15.8 | 5.3 | 7.2 |
| Diethylstilbestrol | 15.304 | 4.635 | 5.659 | 14.8 | 4.3 | 5.0 |
| Dihydroetorphine | 15.670 | 5.194 | 6.097 | 17.0 | 7.3 | 7.1 |
| Diltiazem | 15.540 | 4.979 | 5.959 | 14.8 | 4.3 | 5.0 |
| Doxapram | 15.461 | 4.874 | 5.857 | 14.5 | 2.9 | 4.6 |
| Doxylamine | 15.596 | 5.004 | 6.011 | 17.0 | 7.3 | 7.1 |
| Efavirenz | 15.206 | 4.485 | 5.671 | 15.8 | 5.3 | 7.2 |
| Entecavir | 15.748 | 5.286 | 6.736 | 14.8 | 4.3 | 5.0 |
| Etorphine | 15.782 | 5.299 | 6.137 | 17.0 | 7.3 | 7.1 |
| Finasteride | 15.026 | 4.221 | 5.242 | 14.8 | 4.3 | 5.0 |
| Fluconazole | 15.901 | 5.514 | 6.695 | 14.8 | 4.3 | 5.0 |
| Glipizide | 15.460 | 4.902 | 6.086 | 14.5 | 2.9 | 4.6 |
| Indinavir sulfate | 15.184 | 4.590 | 5.746 | 14.8 | 4.3 | 5.0 |
| Itraconazole | 15.121 | 4.450 | 5.453 | 14.8 | 4.3 | 5.0 |
| Ketoconazole | 15.323 | 4.708 | 5.849 | 15.8 | 5.3 | 7.2 |
| Lacidipine | 14.944 | 4.142 | 5.453 | 14.8 | 4.3 | 5.0 |
| levocetrizine | 15.514 | 4.947 | 5.941 | 17.0 | 7.3 | 7.1 |
| Sitagliptin | 15.648 | 5.185 | 6.132 | 15.8 | 5.3 | 7.2 |
| lorazepam | 15.610 | 5.057 | 6.137 | 17.0 | 7.3 | 7.1 |
| Lornoxicam | 15.092 | 4.369 | 5.760 | 15.8 | 5.3 | 7.2 |
| Methylprednisolone | 15.985 | 5.682 | 6.418 | 14.8 | 4.3 | 5.0 |
| Metoclopramide | 15.635 | 5.154 | 6.196 | 17.0 | 7.3 | 7.1 |
| Metolazone | 16.182 | 5.950 | 6.685 | 14.5 | 2.9 | 4.6 |
| Mitomycin C | 15.597 | 5.111 | 6.294 | 15.8 | 5.3 | 7.2 |
| Mitragynine | 15.478 | 4.965 | 5.969 | 14.5 | 2.9 | 4.6 |
| Naproxen | 16.017 | 5.675 | 6.466 | 14.8 | 4.3 | 5.0 |
| Paliperidone | 15.749 | 5.354 | 6.217 | 14.5 | 2.9 | 4.6 |
| Phenylpropanolamine | 15.942 | 5.551 | 6.533 | 15.8 | 5.3 | 7.2 |
| Pioglitazone | 15.462 | 4.945 | 6.041 | 14.5 | 2.9 | 4.6 |
| Prednisolone | 15.663 | 5.186 | 6.165 | 15.8 | 5.3 | 7.2 |
| Tegaserod | 15.294 | 4.760 | 6.053 | 14.8 | 4.3 | 5.0 |
| Temozolomide | 15.927 | 5.429 | 6.468 | 15.8 | 5.3 | 7.2 |
| Tipranavir | 15.140 | 4.639 | 5.624 | 14.5 | 2.9 | 4.6 |
| Verapamil | 15.697 | 5.366 | 5.901 | 14.5 | 2.9 | 4.6 |
| Vincristine | 15.503 | 5.144 | 5.440 | 17.0 | 7.3 | 7.1 |
| Vinorelbine | 15.223 | 4.749 | 5.363 | 14.8 | 4.3 | 5.0 |
| Zofenopril | 15.366 | 4.850 | 5.902 | 14.8 | 4.3 | 5.0 |

***Table (S7): predicted and reported HSPs of the test set***

| **Drug** | **Predicted HSPs** | | | **Reported HSPs** | | |
| --- | --- | --- | --- | --- | --- | --- |
| Rifampicin | 15.596 | 5.222 | 5.755 | 15.8 | 5.3 | 7.2 |
| Ivabradine | 15.775 | 5.382 | 6.154 | 15.8 | 5.3 | 7.2 |
| Hydroxyzine | 15.585 | 5.077 | 5.945 | 15.8 | 5.3 | 7.2 |
| 5-Fluorouracil | 15.912 | 5.457 | 6.676 | 15.8 | 5.3 | 7.2 |
| Fenofibric Acid | 15.579 | 5.047 | 5.980 | 15.8 | 5.3 | 7.2 |
| Phenobarbital | 15.723 | 5.224 | 6.232 | 14.8 | 4.3 | 5.0 |
| Dolutegravir | 16.066 | 5.761 | 6.632 | 14.8 | 4.3 | 5.0 |
| Nimesulide | 15.770 | 5.310 | 6.296 | 14.8 | 4.3 | 5.0 |
| Isradipine | 15.860 | 5.41 | 6.252 | 14.8 | 4.3 | 5.0 |
| Digoxin | 15.173 | 4.605 | 5.550 | 14.8 | 4.3 | 5.0 |
| Nimodipine | 15.405 | 4.832 | 5.927 | 14.5 | 2.9 | 4.6 |
| Betamethasone | 15.642 | 5.163 | 6.215 | 14.5 | 2.9 | 4.6 |
| Tolfenamic acid | 15.822 | 5.374 | 6.152 | 14.5 | 2.9 | 4.6 |
| Prochlorperazine maleate | 15.072 | 4.403 | 5.503 | 17.0 | 7.3 | 7.1 |
| Ebastine | 15.784 | 5.337 | 6.127 | 17.0 | 7.3 | 7.1 |

***Figure (S1): Chromatograms of the investigated drugs after extraction with the predicted solvents' mixtures***

| 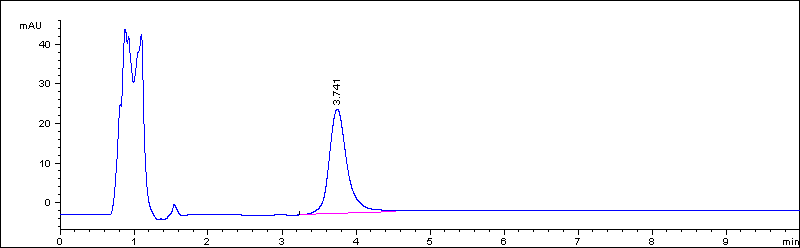 | 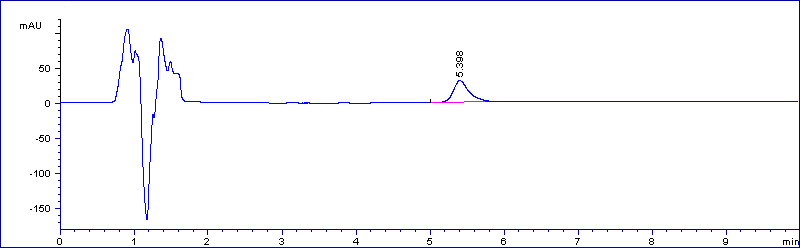 |
| --- | --- |
| Amlodipine | Ambrisentan |
| 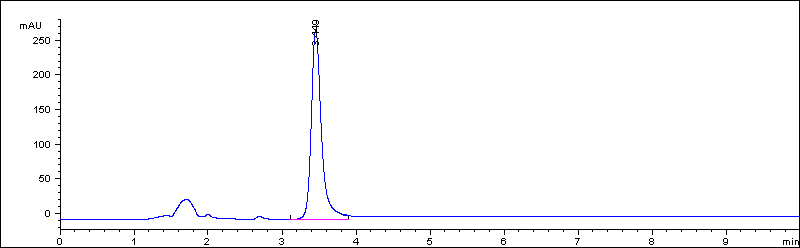 | 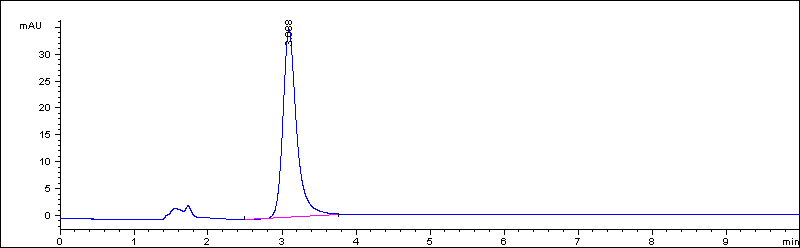 |
| Indapamide | Risperidone |
| 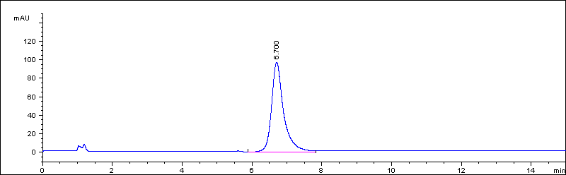 | 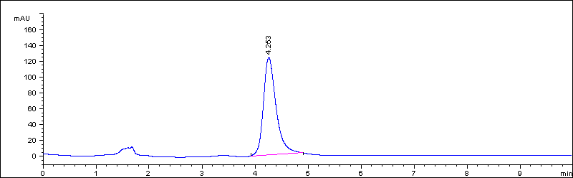 |
|  |  |
| Oxcarbazepine | Desloratadine |
| 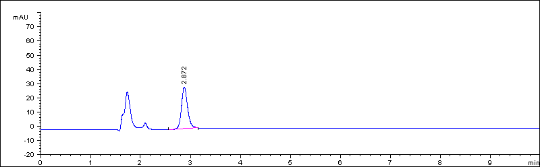 | 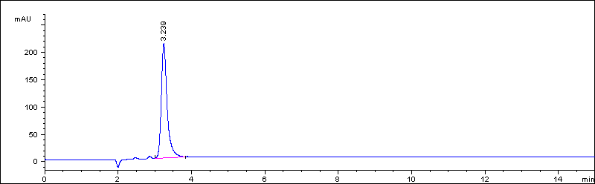 |
| Tenofovir Alafenamide | Moxifloxacin |
| 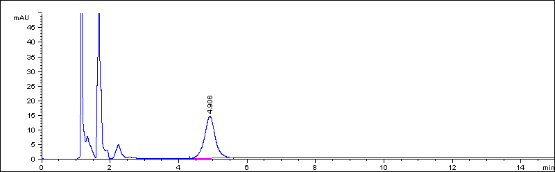 | 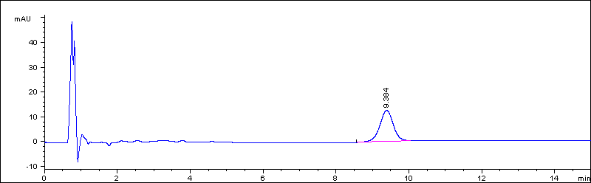 |
|  |  |
| Saxagliptin | Etodolac |
| 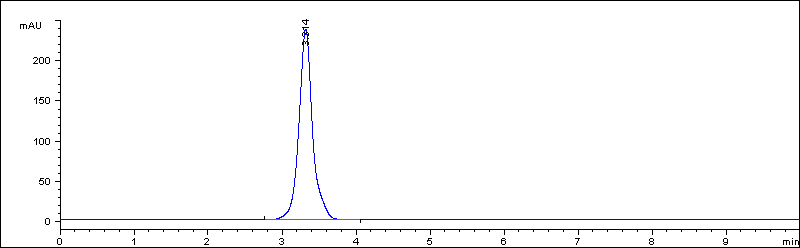 | 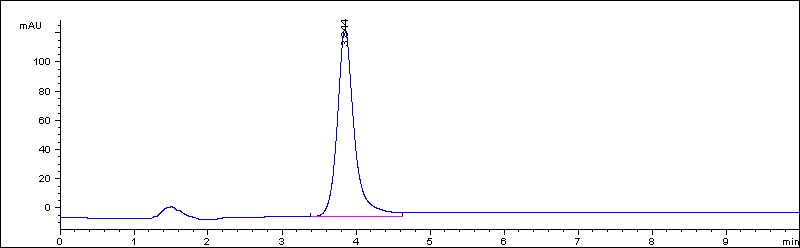 |
|  |  |
| Omeprazole | Escitalopram |
| 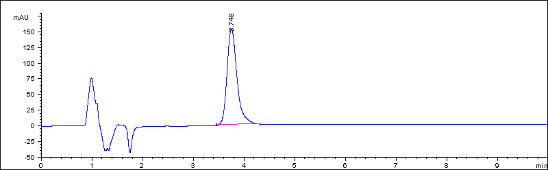 | 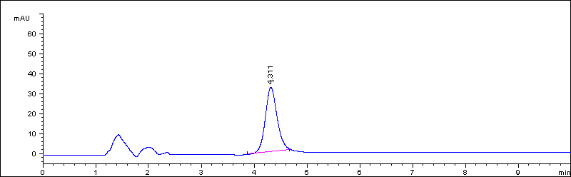 |
| Sertraline | Nebivolol |
| 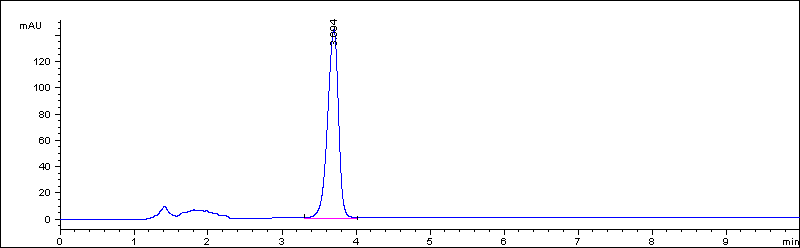 | 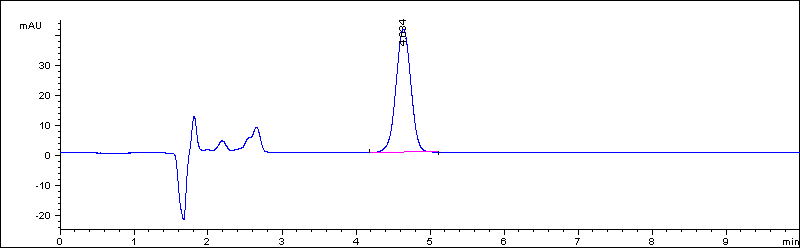 |
| Telmisartan | Donepezil |
| 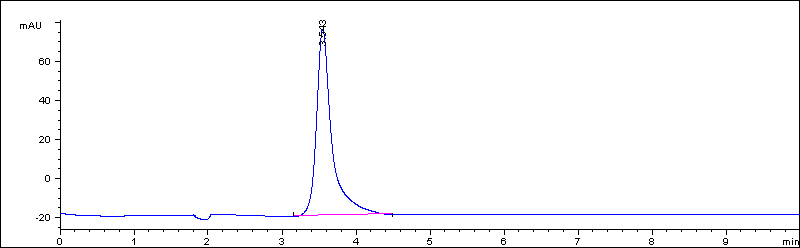 | 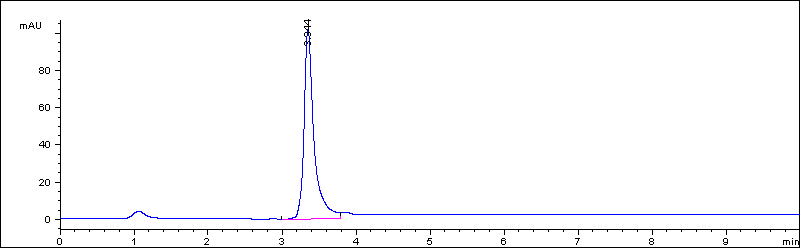 |
| Levofloxacin | Valacyclovir |
| 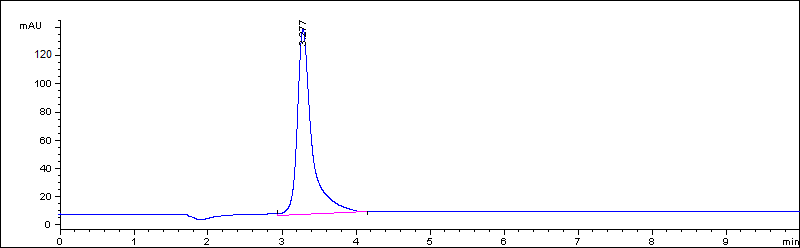 | 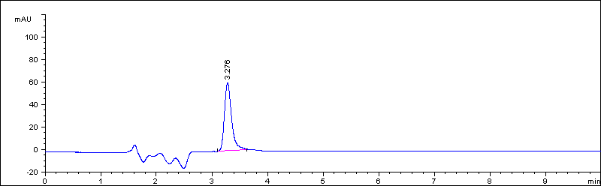 |
| Linagliptin | Chlorpheniramine maleate |

***Figure (S2): Chromatograms of the blank of the investigated drugs after extraction with the predicted solvents' mixtures.***

| 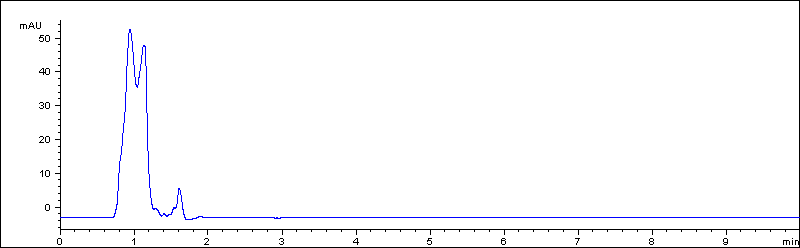 | 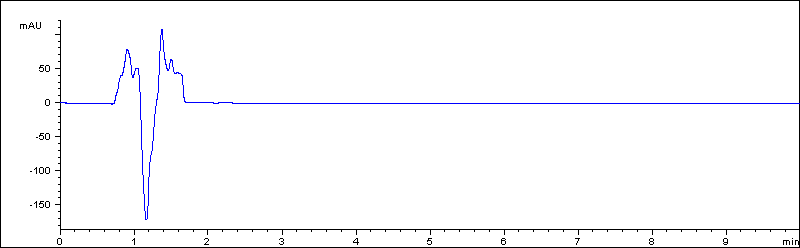 |
| --- | --- |
| Amlodipine | Ambrisentan |
| 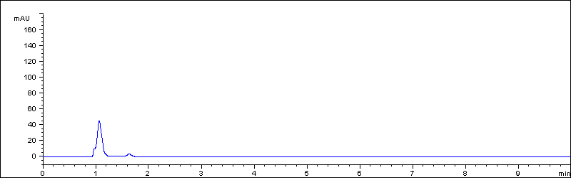 | 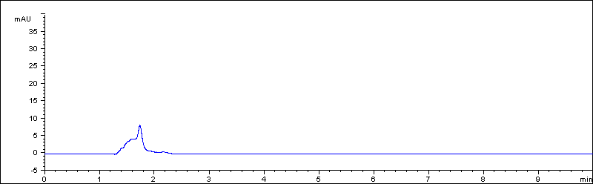 |
| Indapamide | Risperidone |
| 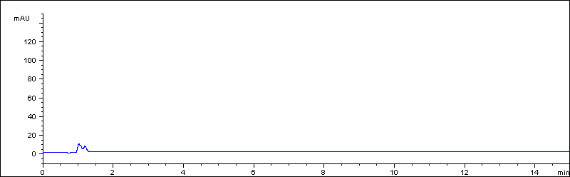 | 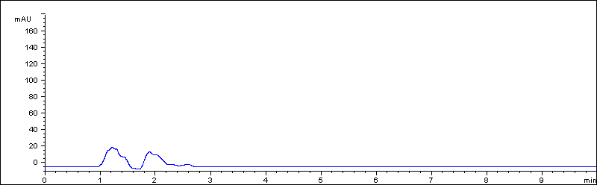 |
|  |  |
| Oxcarbazepine | Desloratadine |
| 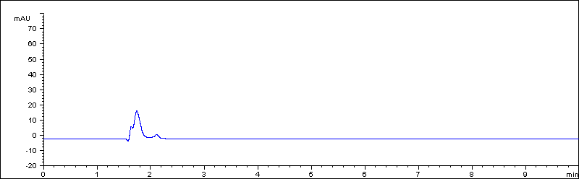 | 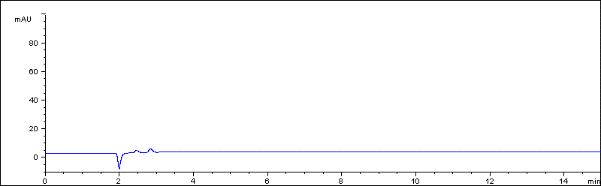 |
| Tenofovir Alafenamide | Moxifloxacin |
| 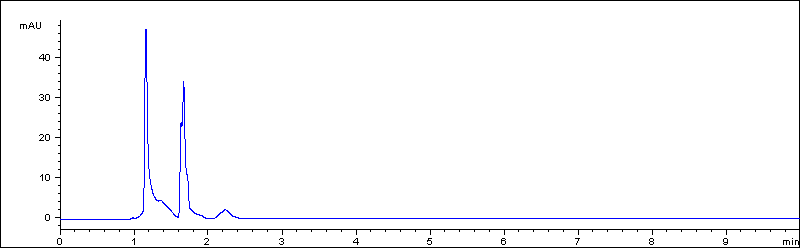 | 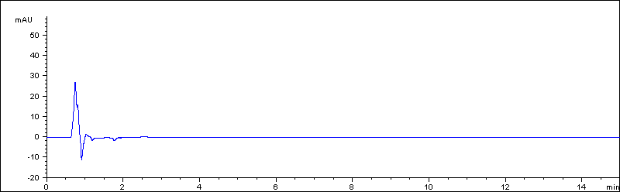 |
|  |  |
| Saxagliptin | Etodolac |
| 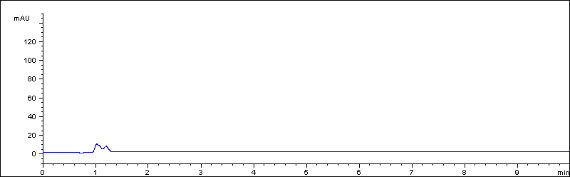 | 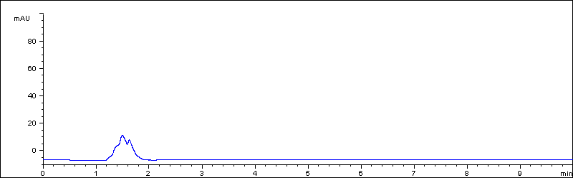 |
|  |  |
| Omeprazole | Escitalopram |
| 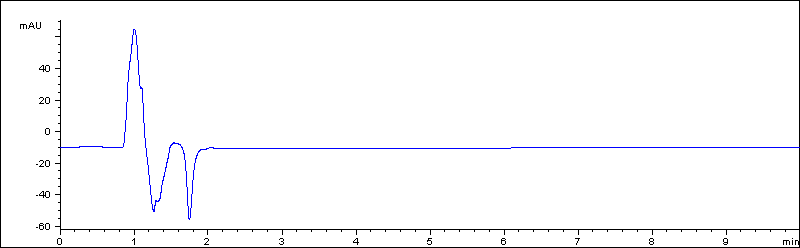 | 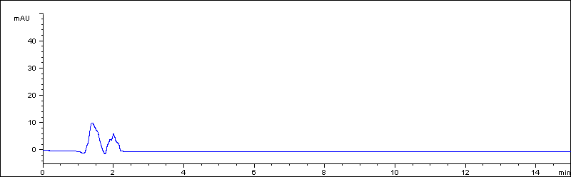 |
| Sertraline | Nebivolol |
| 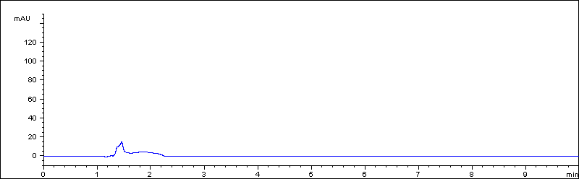 | 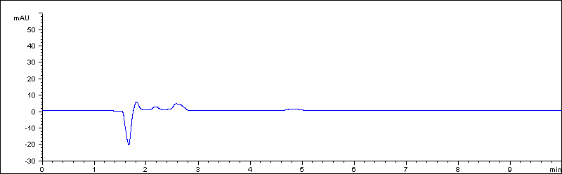 |
| Telmisartan | Donepezil |
| 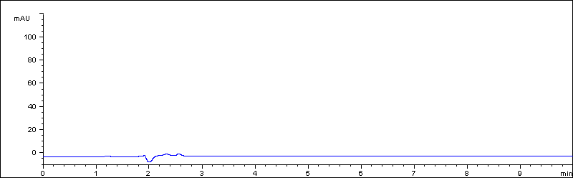 | 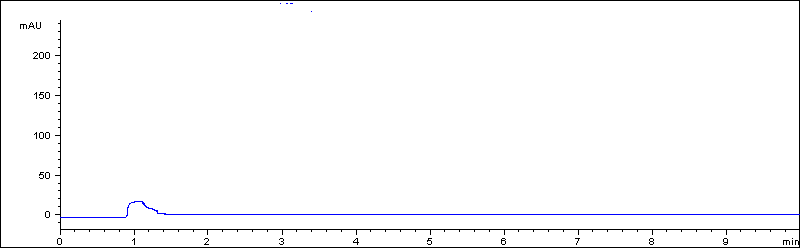 |
| Levofloxacin | Valacyclovir |
| 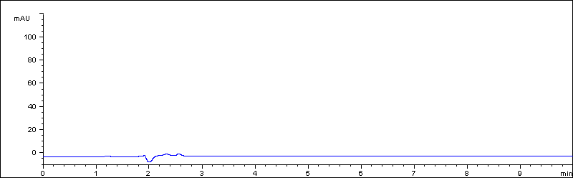 | 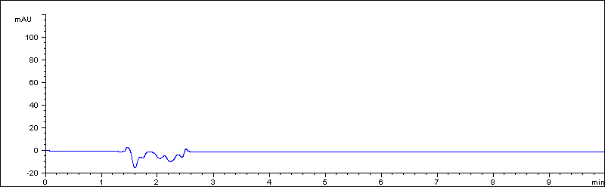 |
| Linagliptin | Chlorpheniramine maleate |
